# How much choice is there in housing choice vouchers? Neighborhood risk and free market rental housing accessibility for active drug users in Hartford, Connecticut

**DOI:** 10.1186/1747-597X-4-5

**Published:** 2009-04-15

**Authors:** Julia B Dickson-Gomez, Ellen Cromley, Mark Convey, Helena Hilario

**Affiliations:** 1 Medical College of Wisconsin, Center for AIDS Intervention Research, 2071 N. Summit Avenue, Milwaukee, WI 53202, USA; 2 The Institute for Community Research, 2 Hartford Square West, Suite 100, Hartford, CT 06106, USA

## Abstract

**Background:**

Since the mid-1970s, the dominant model for U.S. federal housing policy has shifted from unit-based programs to tenant based vouchers and certificates, intended to allow recipients a choice in their housing and neighborhoods. Surprisingly little research has examined the question of where those with Section 8 housing vouchers are able to live, but some research suggests that voucher holders are more likely to reside in distressed neighborhoods than unsubsidized renter households. Further, federal housing policy has limited drug users' access to housing subsidies. In turn, neighborhood disorder has been associated with higher levels of injection drug risk behaviors, and higher drug-related mortality. This paper explores rental accessibility and neighborhood characteristics of advertised rental housing in Hartford CT.

**Methods:**

Brief telephone interviews were conducted with landlords or management companies with units to rent in Hartford to explore housing accessibility measured as initial move in costs, credit and criminal background checks, and whether rental subsidies were accepted. These data were supplemented with in-depth interviews with landlords, shelter staff and active users of heroin, crack or cocaine. Apartments for rent were geocoded and mapped using **ArcGIS**. We used location quotients to identify areas where low-income rental housing is concentrated. Finally, we mapped apartments in relation to drug and violent arrest rates in each neighborhood.

**Results:**

High security deposits, criminal background and credit checks limit housing accessibility even for drug users receiving vouchers. While most landlords or management companies accepted housing subsidies, several did not. Voucher units are concentrated in neighborhoods with high poverty neighborhoods. Landlords reported little incentive to accept rental subsidies in neighborhoods with low crime rates, but appreciated the guarantee provided by Section 8 in high crime neighborhoods that were less likely to attract applicants with good jobs and credit.

**Conclusion:**

Housing vouchers in themselves do not greatly improve recipients' choice of neighborhood and voucher units are concentrated in the most distressed neighborhoods. Policy changes are needed to increase landlords' incentives to accept housing subsidies. Interventions to improve neighborhood conditions are needed to improve the probability of success for those recovering from drug addictions.

## Backgound

Research has shown a strong relationship between substance abuse and homelessness in the United States, Europe and Canada. Substance abuse problems afflict anywhere from 28 to 67% of homeless individuals [1-4] and substance abuse increases an individual's vulnerability to homelessness [5-9]. Given the strong association between substance abuse and homelessness, substance abuse often has been hypothesized as a cause of homelessness [10-12]. Other researchers have argued that the failure of wages from low-skill jobs to keep pace with rising housing costs are the causes of increased rates of homelessness [13,14]. More recent research has combined these perspectives arguing that economic changes have led to a scarcity of low-income housing, but that personal factors, such as substance abuse, explain who is least able to access housing in an increasingly competitive housing market [13,15-18].

In the U.S. national and state policies have affected the location and availability of low-income and subsidized housing. Public housing emerged in the late 1930s as a Depression-era public works program and evolved over time into public housing programs serving poorer and increasingly minority families [19]. Public housing projects were often located in the most distressed urban areas because of neighborhood resistance [19,20]. Since the mid-1970s, the dominant model for U.S. federal housing policy has shifted from unit-based programs to tenant based vouchers and certificates (e.g. the Section 8 or Shelter Plus Care programs) [19-22] in order to allow recipients a choice in their housing and neighborhoods. Surprisingly little research has examined the question of where those with housing vouchers are able to live. Some studies have found that housing voucher holders are less likely to live in distressed neighborhoods than public housing residents [23,24]. Other research has found that voucher holders are more likely to reside in distressed neighborhoods than unsubsidized renter households [20,23], and that African American voucher holders are more likely to live in distressed neighborhoods than White voucher holders [20].

There are several potential explanations for the continued concentration of voucher holders in distressed neighborhoods. There may be an inadequate supply of low-cost rental housing in private control. Also, low-cost rental housing may be concentrated in distressed neighborhoods, while some more affluent communities may have housing markets dominated by single family homes [20]. Landlords may not be willing to accept housing vouchers, particularly in tight housing markets with high demand and low vacancy rates [20]. Finally, housing vouchers alone do not eliminate racial segregation and discriminatory renting [20,21,23]. In turn, neighborhood disorder (measured by poverty, vacant buildings, vandalism, trash, drug selling, and burglary) has been associated with higher levels of injection drug risk behaviors, and higher drug-related mortality [25-28].

While the federal government provides rental assistance to approximately 4.6 million low-income renters, more than twice as many (9.7 million) receive no federal housing assistance [29]. Drug users are even less able to access vouchers than non-drug using low-income residents. The federal "One Strike and You're Out" law (P.L. 104–120, Sec.9), passed in 1996, allows federal housing authorities to consider drug and alcohol convictions of subsidy recipients and their family members when making decisions to evict them from or deny access to federally subsidized housing. Drug users are less likely to obtain employment, further limiting their access to housing [30]. Even those few drug users who are able to access housing vouchers face additional barriers when trying to access free-market rental housing including criminal background and credit checks, and high security deposits. In response to these recognized problems, supportive housing has been proposed as a way of increasing housing access and stability among the chronically homeless, including substance abusers. Supportive housing is permanent, subsidized housing with supportive services such as intensive casework, substance abuse and mental health treatment [31]. Whether these programs allow recipients a choice in their neighborhoods is a question in need of research.

Little research has documented drug users' attempts to access free market rental housing, and less still has included landlords' perspectives on apartment application procedures, their willingness and motivation to accept rental subsidies, and their preferences for tenants. In this exploratory paper, we examine the locations of apartments advertized for rent in September, 2004, in Hartford Connecticut. Hartford is a small city, of approximately 18 square miles (47 square kilometers), with a poor and primarily ethnic minority population estimated at 124,848 people, 30% of whom live below the federal poverty line, and 72.7% of whom are non-white race[32]. We also explore the affordability and accessibility of apartments for rent in terms of their monthly rents, security deposits, application fees, criminal background and credit checks, and levels of neighborhood risk. Finally, drawing from in-depth interviews with landlords and drug users, we explore incentives and disincentives to accepting housing subsidies, other barriers faced by drug users in accessing rental housing and the effects of neighborhood drug and violent crime on drug using residents.

## Methods

### Identification of available rental units

In September 2004, apartments for rent in Hartford were identified by reading newspaper advertisements, searching online, and conducting a windshield survey of neighborhoods. In essence, this methodology replicates the steps an individual searching for an apartment would need to take and is, therefore, an appropriate methodology for assessing the availability of free market rental housing at a particular point in time. A list of 80 contact telephone numbers was generated from the one-month search.

Each telephone number identified from an advertisement or sign was called a minimum of five times or until the person in charge of renting the unit was located. We conducted brief telephone interviews with landlords or management company representatives with units to rent in Hartford. We asked about the monthly rent of available units, the amount of security deposit required, whether housing vouchers were accepted, the amount of application fees, required background checks on applicants, and whether applicants were required to pay the costs of the required background checks. Of those contacted, 11% (9) refused to be interviewed. The remaining 15 were not contacted in spite of repeated attempts. Interviews were completed for 61% (54) of the numbers listed.

### Analysis of rental unit characteristics

Data from the brief telephone interviews were analyzed in SPSS for descriptive statistics including number of available units, and the size and location of each available unit. Several landlords and apartment managers had more than one unit on a property available for rent, resulting in a total of 74 apartment units. Data were collected for each available unit. Affordability and accessibility of apartments for rent were assessed by computing average monthly rents for units of different sizes (one-bedroom, two-bedroom, etc) average security deposit for each unit size, and total initial costs for renting each unit (security deposits and application fees). We also assessed accessibility by documenting whether criminal and credit background checks were required and whether or not the costs were paid by the applicant. Means, medians, range and standard deviations were calculated for continuous variables (monthly rents, security deposits and application fees), while frequencies were calculated for nominal data (whether or not criminal background and credit checks were required). The addresses of the units were also recorded. Apartments for rent were address-match geocoded and mapped using ArcGIS 9.2.

To characterize neighborhoods where available housing units were found, data on the number of people living in poverty and the number of housing units that were rental units were collected at the block group level from the 2000 Census of Population and Housing [32]. The block group level was chosen because these units can be aggregated to the city's 17 neighborhoods. Location quotients were calculated to identify neighborhoods where people living in poverty and low-income rental housing are concentrated.

The location quotient is a ratio of ratios used to quantify the degree of relative concentration of an activity in an area. It is a measure used in economic and social geography and has also been adopted in health research [33,34]. In the case of poverty, the ratio in the numerator is the number of people in a Hartford neighborhood living in poverty divided by the total population of the neighborhood. This ratio is then divided by the number of people living in poverty in the city of Hartford divided by the total population of Hartford. In the case of rental housing, the ratio in the numerator is the number of rental units in the neighborhood to the total housing units in the neighborhood. This ratio is then divided by the number of rental units in the city of Hartford divided by the total number of housing units in Hartford. Ratios greater than 1 indicate a higher concentration of poverty or available rental housing in the neighborhood than in the city as a whole, while those under 1 indicate a low relative concentration of poverty or rental housing. For example,



where,

LQ_i _is the location quotient for neighborhood i

e_i _is the number of rental units in neighborhood i

e is the number of all housing units in neighborhood i

E_i _is the number of rental units in Hartford

E is the number of all housing units in Hartford

Data on the number of drug-related and violent crime arrests were obtained from the Hartford Police Department. These data were used to identify neighborhoods with higher concentrations of these types of crimes. The locations of available apartments were mapped against the concentrations of poverty, rental units, and drug and violent arrests in each neighborhood.

### In-depth interviews with landlords, service providers, and active drug users

During the brief telephone interviews, landlords were asked whether they would be willing to participate in an in-depth interview regarding their experiences renting to tenants with federal housing subsidies and in renting to drug-using tenants. Of those who completed a brief telephone interview, 14 agreed to be contacted for an in-depth interview. Due to difficulties in scheduling, only 7 in-depth interviews were conducted with landlords or apartment managers. Those who agreed to an in-depth interview were more likely to have cheaper rents and accept housing subsidies than those who did not agree to an interview. One of the seven managed a property that did not accept housing subsidies, while the other six accepted tenants with housing subsidies. Landlords and apartment managers were asked about their application procedure, specific problems they have faced with drug using participants, their experiences with housing subsidies and the organizations that administer them, and the incentives and disincentives to accepting housing subsidies.

In addition to the interviews with landlords, we conducted key informant interviews with twelve service providers including shelter, supportive housing, and substance abuse treatment staff in order to obtain service provider perspectives on the barriers and facilitators drug users face in accessing information, housing and services. Key informants were purposefully sampled to represent the variety of service providers who come into contact with homeless drug users. A list of service providers was developed through internet searches, project team members' knowledge of social services in the area, and recommendations from interviewed key informants. Key informants represented staff in different positions within their organizations including the executive director of one organization, supervisors and caseworkers with more direct, daily interactions with clients. Participants were 60% female, 60% white, 30% African American, and 10% Latino. The refusal rate among service providers was approximately 50%. Most refusals were due to time constraints or scheduling problems.

In-depth interviews were also conducted with 65 active drug users at baseline, 3- and 6- months. Because this paper focuses on neighborhood characteristics and the accessibility of rental and subsidized housing, interviews are analyzed as cross-sectional data. Eligibility criteria included being over 18 years old, and having used crack, cocaine or heroin in the last 30 days. Participants were purposefully sampled to reflect drug users in a variety of housing situations including homeless on the street (n = 4), homeless in shelters (n = 14), temporarily doubled up with family or friends (n = 11), or independently housed in subsidized (n = 10), supportive (n = 4) or free market rental housing (n = 10).

Participant recruitment for the drug using sample was achieved through a combination of direct street recruitment and referral from other projects. For participants who were directly recruited, we targeted recruitment in locations where populations of drug users with differing housing characteristics could be found. Drug users who were homeless were recruited from each of Hartford's seven shelters or soup kitchens. Outreach staff approached potential participants in these settings, distributed HIV prevention materials such as bleach kits and condoms to initiate a general discussion about risk behaviors and assess their general eligibility for the study. Those participants who appeared interested and eligible were given an appointment card for full screening. Drug users who were doubled up with family or friends or housed in subsidized, non-subsidized or supportive housing were similarly recruited through street outreach, or from prior knowledge of their situation from ethnographic research in other research projects working with active drug users. We attempted to recruit equal numbers of drug users (approximately 10 or 11) from each of the housing statuses. In practice it was much easier to recruit participants in some housing categories than others (e.g. homeless in shelter and participants doubled up with family or friends were easier to identify and recruit than homeless on the street or drug users in supportive housing). Drug users were asked to describe their housing histories, current housing situation, experiences applying for housing subsidies and social service benefits. Those who were currently looking for an apartment were asked to describe their experiences applying for apartments.

Verbal consent was obtained from landlords and apartment managers who participated in the brief telephone interviews. Written informed consent was obtained from all participants in the in-depth interviews prior to interviewing. Drug using participants were compensated $25 for each interview completed. The Institute for Community Research's Institutional Review Board reviewed and approved study protocol and consent procedures.

All in-depth and key informant interviews were transcribed verbatim and analyzed for key themes using Atlas.ti qualitative analysis software. Interviews were first coded for participant demographics, type of interview (landlord, key informant, or drug user) and, for drug users, housing status at the time of the interview. Interviews were then coded a second time for content, using broad topic areas such as apartment application experiences, landlord preferences, and subsidy application experiences. Interviews were then coded a third time to refine categories and explore emerging themes. For example, "landlord preferences" was further refined to include stigmatizing beliefs toward subsidy recipients, incentives to accept housing subsidies and disincentives to accept housing subsidies. The coding scheme was developed by coding the first several interviews in an iterative and collaborative process by the first, third and fourth authors until all team members felt that the coding tree reflected the data and was easily understood. Thereafter, coding was done by the third and fourth authors and reviewed by the entire team. Disagreements were discussed until consensus was reached.

## Results/Discussion

Of the 65 participants interviewed at baseline, 46% were African American, 46% Puerto Rican, 8% white, and 46% were women. The mean age was 43 years (s.d. 6.8 years). Those who smoked crack smoked a mean of 39 times in the prior 30 days (s.d. 45), while those injecting heroin injected an average of 32 times (s.d. 3.4). Participants were low income with 63% having earned less than $500 in the last 30 days and 94% having earned less than $1000. Twenty-six percent of our sample received housing subsidies at baseline. Although we purposefully sampled to include drug users who were currently housed, 83% of the sample reported being homeless at least once in their lifetimes, 40% of the sample moved at least once during the study period, and 12% had moved four or more times, indicating a high degree of housing instability. Interviews with landlords included three who were managers of several properties, and four who were owners of their rental properties. Two of the seven landlord interviews were with women.

Table 1 shows the mean and range of monthly rents for different sized units, the number of units available, and the average security deposits required for each sized unit. The average monthly rent for each unit size greatly exceeds the income of participants and there were few efficiency units available. In addition, 93% of the units required criminal background checks, and 86% required participants to pay for credit checks. Credit check costs ranged from $20 to $50. Seventy-two percent of the units required criminal background checks.

**Table 1:** Monthly rents of apartments advertised for rent

	Number of available units	Mean monthly rent	Range	Mean security deposit
Efficiency	5	$580	$495–$725	$711
1 bedroom	30	$682.75	$450–$1500	$883
2 bedroom	26	$893.75	$500–$2500	$1221
3 bedroom	11	$870.45	$700–$1100	$1141

4 or more bedrooms	2	$1050	$1000–$1100	$1300

### Location of Subsidized and Unsubsidized Housing

We were able to map 41 addresses of buildings with available rental units for which we had data from the brief telephone interviews. One landlord refused to provide any information on the location of the units available. Ten other landlords, including some with apartments at different locations, would only identify the names of the streets where the buildings with available units were located and this did not provide enough information to geocode the units

Some differences were observed between the apartments in building that could not be geocoded and the apartments in buildings that were geocoded. Overall, the apartments that could not be geocoded were less expensive, more likely to require criminal background checks, and more likely to accept subsidies. Average monthly rent for a one-bedroom apartment in a building not geocoded was $589 and the average total first month cost including rent, security deposit, and credit and criminal background checks was $1,264. Geocoded one bedroom apartments had an average monthly rent of $699 and an average total first month cost of $1,520 due to higher security deposit requirements. The buildings that could not be geocoded were much more likely to require criminal background checks (91%) than buildings that were geocoded (61%). Nine of the 11 buildings (81%) that could not be geocoded were places where landlords accepted subsidies while only 73% of the buildings that could be geocoded accepted subsidies.

**Figure 1:**
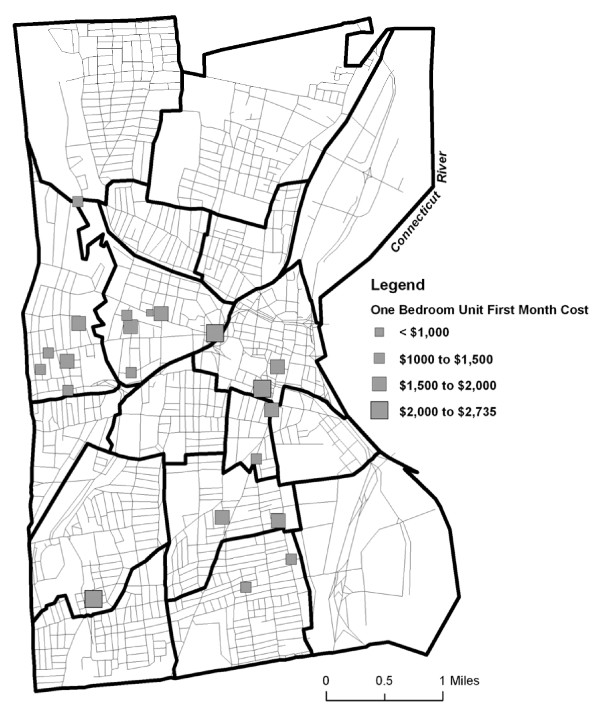
**Unit locations with subsidy available related to concentration of people living in poverty**.

Figure 1 shows the location and initial move in costs including first months rent, security deposit and application fees for those apartments that could be geocoded.

**Figure 2:**
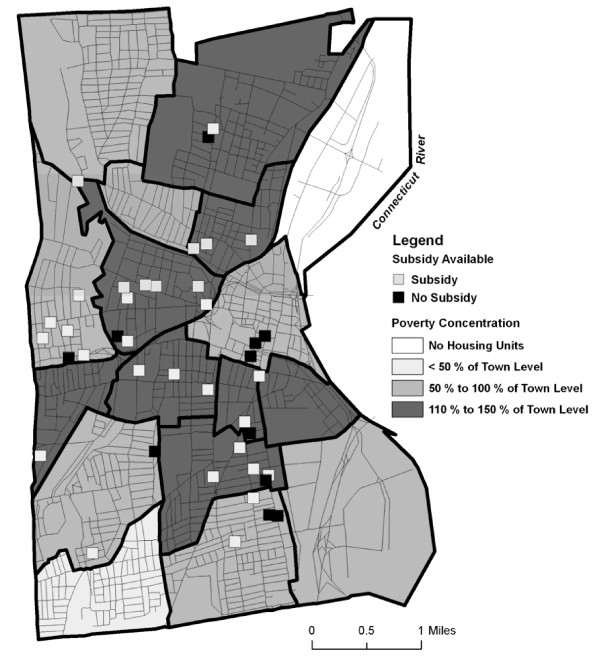
**Unit locations with subsidy available related to concentration of rental units**.

Those apartments that did not accept subsidies tended to be located in neighborhoods with lower concentrations of people living in poverty (Figure 2). An exception occurred in the West End neighborhood of the city where five of the six buildings accepted subsidies and the concentration of rental housing was low relative to the city as a whole.

**Figure 3:**
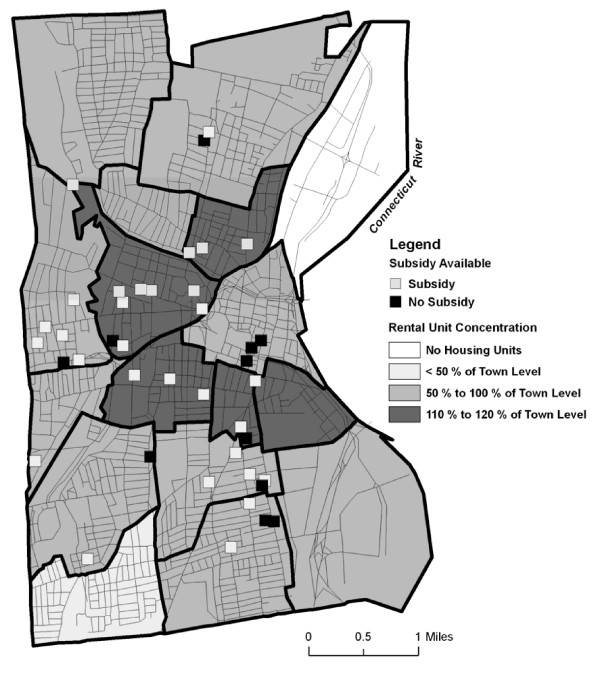
**First month's cost for a one-bedroom apartment including first month rent, security deposit, and application fees**.

Approximately half of the buildings with apartments that accepted subsidies were located in neighborhoods that had high concentrations of rental housing relative to the city as a whole (Figure 3). In contrast, only two apartments that did not accept subsidies were located in neighborhoods that had high concentrations of rental housing relative to the city as a whole. Most apartments that did not accept subsidies were located in neighborhoods with relatively less rental housing.

**Figure 4:**
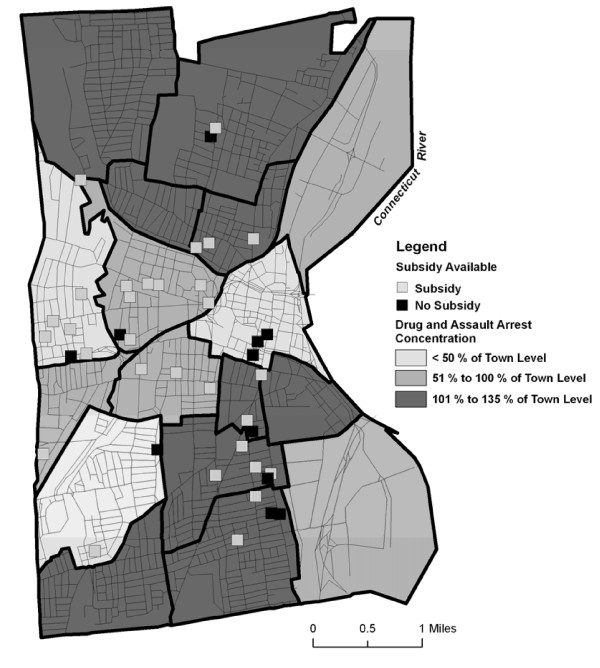
**Number of apartments available for rent in neighborhoods with different concentrations of drug and assault arrests**.

Most advertised units that could be geocoded were not located in neighborhoods with high concentrations of drug arrests and violent crimes (Figure 4). About 45% of buildings with apartments for rent were in high crime areas. This percentage was about the same for buildings that did and did not accept subsidies.

### Incentives and Disincentives to Accepting Housing Subsidies

Interviews with landlords or apartment managers provide insight into the incentives or disincentives to accept housing subsidies, and the influence of neighborhood characteristics on landlord's decision making processes. One disincentive to accepting housing subsidies is the negative perception landlords have of people who receive subsidies as described by the apartment manager below.

Ethnographer: So, what type of housing subsidies do you accept here?

Joe (Apartment Manager): They accept nobodies' anymore...No Section 8, no nothing anymore. Been that way now for a year...It makes for a better building. The people that are Section 8 people, not all of them, don't get me wrong... but the majority don't work so it makes for a rowdy building...Everybody here now works. I do have Section 8s in here, but they have been here for a long time...What really hurts me the most is because you have a lot of people that's let's say 50 years and out that are now going on Section 8. Their hell days are over... They would make a good tenant. But the owners just decided not to take any more. We used to do them all...every organization at one time.

The apartment manager expresses a number of stigmatizing beliefs regarding Section 8 recipients, i.e. that they do not work, and are "rowdy" implying that they may have lifestyles including substance abuse that make them undesirable tenants. He makes a distinction between older Section 8 recipients, who may be unable to work due to health problems and have fixed incomes upon retirement, and younger Section 8 recipients. The apartment manager does not acknowledge that Section 8 is a housing subsidy, and thus provides no income, or that many employed in low-income jobs cannot earn enough to pay for free market rental housing.

In addition to negative attitudes toward Section 8, landlords mentioned that another disincentive to accept housing subsidies was in having to pass the housing inspections.

Karen (landlord): I guess because I'm a landlord I'm considering it nit picking, but they're very picky about how they want the apartment to be and I guess that's for the safety of the tenant. I find it very difficult though, when they want to come in for a re-inspection because they hold the money. If the apartment fails they hold the rent and so you kind of want to get them back in there as quickly as possible and that's when I find they kind of can put you on hold for a bit, for the re-inspection, and then you're without your rent...In one apartment the tenant chose to remove the stove knobs for her children's sake and he [the inspector] failed the apartment because of that. And, I'm like, well, the parent chose to do that, that's not something I did.

Although safety inspections help ensure a minimum of safety and quality in Section 8 recipients' housing, the rules they enforce can sometimes act as barriers to housing access when property owners and managers grow frustrated with Section 8 guidelines.

While stigmatizing attitudes toward Section 8 recipients and negative experiences with home inspections are probably shared by many landlords, other interviews suggest that neighborhood characteristics play a role in decisions about whether or not to accept housing subsidies. In higher income and lower-crime, more desirable neighborhoods, landlords may have the luxury of being choosy about their tenants, limiting them for example to only those who are employed in well-paying jobs. In lower-income neighborhoods with high violent and drug crime, landlords described that being guaranteed a portion of the rent each month was a significant incentive to accept housing subsidies.

Ethnographer: Are there any benefits to accepting Section 8?

Carl (apartment manager): Well, there's pros and cons...We are guaranteed a certain amount from Section 8 every month which is a good thing...There's more paperwork of course because Section 8 is always more paperwork. In some ways it helps the landlord because if the client doesn't follow the rules and regulations of the building or doesn't keep the maintenance of their apartment, you have a resource to go back to their social worker at Section 8 and say, "Look it.... This is the reason I'm putting these people under eviction." I gave them a copy of the eviction notice as well. And sometimes they will notify the tenant...so you kind of have a little more leverage with the tenants that are under Section 8 'cause they don't want to lose it. A lot of these people waited many years to get on...Section 8.... It also stops, like when you have Section 8 programs,... [if] somebody decides to move in with that particular tenant, they are breaking their lease. So again, you have more control in that respect.

While listing disincentives to accepting Section 8, Carl clearly feels that the benefits outweigh the costs. In many respects he seems to expect troublesome tenants, e.g. that they will fail to maintain their apartment or will let other people will move in, and feels that the extra leverage provided by the threat of losing the subsidy allows him to deal with problems without going through lengthy and expensive eviction procedures. Again, this kind of assistance may be more welcome in impoverished neighborhoods with high crime rates, as Carl describes below.

Ethnographer: Have you seen the process for [apartment] applications change over time?

Carl: Well at one time I think they were more stringent than they are today. The market in the Hartford area especially is not as good as it used to be... There are a lot more apartments becoming available which means that either people are moving out of Hartford and going someplace else, and I don't think they are building new apartments (laughs) so something is happening to the flow of people... A lot of people don't like to live here in Hartford. There is a lot of crime in Hartford.

Hartford lost 15% of its population in the last decade [4]. Much of the recent exodus has been of low-income residents to nearby suburbs. A recent study by the Brookings Institution suggests that the exodus of low-income residents to the suburbs is a national trend and that there are now more impoverished residents in suburbs than in the nation's cities [35]. This exodus may have been especially great in the higher poverty neighborhoods. The downtown area of Hartford, on the other hand, has seen increasing business and residential development over the last decade. Much of the residential development has catered to higher income young professional and "empty nesters", i.e. upper middle class suburban professionals whose children have left home [36]. Landlords in higher poverty neighborhoods, therefore, find fewer qualified tenants making those who receive Section 8 relatively attractive.

### Credit checks and security deposits

Initial costs, poor credit reports and histories of criminal conviction constituted significant barriers to accessing housing among participants. Even for those participants who receive housing subsidies, initial move-in costs can limit housing access. Section 8 will not pay for security deposits, or for apartment application fees.

Some participants who received housing subsidies also reported that histories of arrest and eviction were barriers in finding units to rent. Seventy percent of the sample reported having been arrested prior to the study period, while 21% reported being arrested during the 6 month study period. Forty-four percent reported that they had been evicted during their lifetimes.

Ethnographer: Last time you were talking a little bit about sometimes it's hard to get a place because of your record?

Jose (Puerto Rican male heroin user, 42 years old): Yes, oh yes, yes, yes. It's very hard...First thing, let's say you got $1000 for an apartment. It's $20 first for an apartment fee. So now you don't have the $1000. How about if you take five different applications?...That's $100...So now you have $900. Okay then they say, "No you lost your $20." The police record thing, it's true. They look at your record. If you got a record, boom, they don't give you the apartment.

The only participants able to obtain free market rental housing in this study, reported that they personally knew the landlords who were willing to waive background checks, or had someone intervene with a landlord on their behalf such as family members with personal relationships with a landlord, or supportive housing caseworkers [37]. For example, Jose who described difficulty obtaining free market rental housing was able to obtain an apartment almost immediately after receiving supportive housing at the end of the study period. When asked if they did a background check as part of his application, he responded "Yes, but they were pre-advised of my record." While none of the landlords interviewed in this project had any experience with supportive housing programs, a caseworker at one of the supportive housing programs stated,

Charlene: I find it very easy to get them housing because...even though the clients sign the lease, they are not fully responsible... The landlord looks at hey, this is an agency.

Like Carl who felt that he was able to receive assistance from Section 8 caseworkers if he had a problem with a client, this caseworker felt that landlords were willing to accept drug using clients with criminal histories because they knew that the agency offering supportive services would intervene with clients when problems occurred.

### Neighborhood Drug Activity

As seen in the maps above, 45% of geocoded rental units were located in areas with high levels of drug and violent crime. Participants reported that living in areas of high drug use and violence decreased their sense of security, and increased or made abstaining from drug use more difficult.

Ethnographer: What are some of the problems you have encountered [in your neighborhood]?

Maria (Puerto Rican female heroin user, 54 years old): The drug trafficking. I want to quit and I have them [people who are selling] right there...They [drug users] mug people so they can get their fix with the money...Sometimes people see me and they tell me "Come here." They [drug users from outside the neighborhood] send me to buy for them and I go so that they won't get robbed...Sometimes they give me $10 or $20 so I can buy me a bag...That's no life... I think to myself, "God, what kind of life is this?"...

Ethnographer: So, how many people do you think sell drugs in your building?

Maria: The majority of them. There is only about five people out of everyone that don't do it, because the majority of people there sell drugs...Sometimes I think to myself, I want to go detox but what for, the same thing will happen there. If I am going to go in detox then I have to be at a place free of drugs.

Many participants described being asked daily by drug dealers if they would like to buy.

Ethnographer: Now, what do you think of the area around you?

Carmen (Puerto Rican female, 38 years old): I don't like it to be honest with you. Too many people hang around selling drugs... That's all they do all day. I don't like it...for me, because I'm trying to stay clean, you know, so...I have people that tries to like, "You straight? You straight?" ...I get very angry... If I'm not asking you for it then obviously I don't want to buy none, you know? Why you got to approach somebody?...I find that very offensive.

Participants reported that supportive housing programs, both fixed-site where all clients are located in the same building facilitating the delivery of services, and scattered-site where tenants are able to choose their own rental housing, were also often located in high risk neighborhoods. Ray who had been working with a shelter caseworker and was on the waiting list to obtain supportive housing described his reluctance to accept housing in a particularly problematic fixed site building.

Ray (African American male, 45): If they are going to stick me in that apartment building on top of the hill, you know... the white one, crack infested building...One of the guys, he was staying at the shelter for a couple of days, then I found out the police kicked in his door and he...was down at another shelter with us. You know who was living up in his house? Joe and Bob the crack dealer, ran him out of his own house.

Ethnographer: That's supposed to be supportive housing, isn't it?

Ray: Yes ma'am...They give me ...an apartment in that building I'm going to give the key back. I do not wish to live in that building. I'm trying to stay away from the crack. How can I stay away from the crack and my next door neighbors are crack dealers? Or a crack user across the hall?

While the shelter administering the supportive housing building made considerable efforts to decrease crack use and sales in the building, and participants reported that it was much improved, they could not decrease drug use and sales in the neighborhood surrounding the building which remained an area of high drug sales.

In contrast, those who lived in the West End reported being able to limit their drug use. For example, Jose describes having been able to stay clean when living on the West End in contrast to living in a shelter in a high drug and crime area.

Ethnographer: Is there anything you like about that area? Like if you were going to look for your own place to live, would you live there?

Jose: No...If you're an addict you will always be an addict if you live in that area...You would still use...because of the atmosphere and the people, basically the drug dealers. So many of them, everywhere you look there's one of them. One on every corner there's a drug dealer. Walking by it everyday, seeing it, makes you fall. Even if you don't look for it, it's right there.

Ethnographer: So where would you try to find a place?

Jose: Away from that atmosphere.

Ethnographer: Would you go back to the West End?

Jose: Yes. I was clean [in that area] believe it or not...I was clean. It was a clean atmosphere. There's hardly no drugs in that area. So everybody up there respects and likes me.

Other participants, who obtained supportive housing in the West End or in nearby suburbs during the study period, reported that while they continued to use, they were able to decrease their drug use compared to when they lived in drug infested areas of the city. They reported walking to old neighborhoods when they wished to buy or use drugs and not giving their drug using acquaintances their new addresses.

## Conclusion

Results from this study, while qualitative and exploratory, suggest that vouchers do not in themselves greatly increase recipients' choice of neighborhood, and do not eliminate all barriers to accessing housing for drug users. There is a shortage of low-income rental housing even in cities with high concentrations of poverty like Hartford. Initial costs for renting an apartment averaged more than $1,000 for almost all apartment unit types, including efficiencies, greatly limiting accessibility to low-income drug users, the majority of whom earned less than $500 a month. While receipt of housing vouchers can improve housing accessibility in general, tenant based voucher programs do not pay for these initial costs. Criminal background and credit checks, common in most apartment applications, further limit drug users' housing accessibility, most of whom had histories of arrest and eviction.

Not all landlords accept housing vouchers. All landlords interviewed recognized incentives and disincentives to accept housing subsidies. Disincentives included the apartment inspections and increased paperwork associated with housing voucher programs and negative beliefs regarding Section 8 recipients, i.e. that they are drug using, problem tenants who do not work. Incentives included a guaranteed portion of the rent each month. These incentives outweighed disincentives to accepting housing subsidies in low income neighborhoods with high concentrations of rental property, and violent and drug related crime. Indeed, landlords who accepted housing vouchers were more likely to have units in low income neighborhoods with higher concentrations of rental property. Given the hassles associated with Section 8 and other housing subsidies, prejudices against Section 8 recipients, and a lack of any real incentives or sanctions associated with accepting or not accepting Section 8, landlords who can rent to high income, employed individuals with good credit, will do so.

Forty-five percent of geocoded rental units were concentrated in neighborhoods with high drug and assault arrest rates. Participants reported that living in such neighborhoods made decreasing or abstaining from drug use nearly impossible. Their descriptions also shed light on the processes that may explain the observed relationship between neighborhood disorder and higher drug fatalities and injection risk behaviors. More chaotic drug use may decrease drug users' willingness and ability to control or decrease drug use, or use harm reduction practices such as injecting with new syringes, or bleaching used syringes. Perhaps more importantly, drug infested neighborhoods may decrease residents' motivation to reduce the risks associated with drug injection or enter drug treatment as they expect that their efforts will be unsuccessful, and find it impossible to envision a meaningful and productive future for themselves. Indeed, recent research has shown that the association of neighborhood disorder with drug use and high-risk sex is often mediated by psychological distress [38,39].

The results of this study suggest a number of structural interventions to increase the housing access of drug users and decrease their drug use. The number of housing subsidies available should be increased to meet the demand. The ban on convicted drug felons receiving housing subsidies should be eliminated. Landlords, however, are often unwilling to rent to applicants with criminal convictions or poor credit, even if these applicants receive housing subsidies. Supportive housing programs have been offered as a promising solution to chronic homelessness and may decrease some of the barriers drug users face in accessing housing. In this study, participants who received supportive housing were often able to receive free market rental housing in spite of criminal histories, due to the intervention of their supportive housing caseworkers. These caseworkers could often act as mediators in landlord and tenant disputes, and intervene with tenants to address problems before they were evicted [40,41].

A growing body of research has shown that providing stable housing through housing subsidies or supportive housing reduces HIV risk behaviors and drug use, decreases use of emergency and inpatient health services, increases anti-retroviral medication adherence, and improves mental health as compared to homeless participants placed on wait-listed control conditions [42,43]. There is also a growing body of literature demonstrating a link between neighborhood characteristics, such as crime, drug use and sales, and perception of neighborhood amenities and opportunities, with poorer health outcomes and increased drug use [26,38,39]. Evaluations of programs to move low-income residents from distressed to better neighborhoods have shown improvements in the mental and physical health of residents who moved to better neighborhoods compared to those who remained in distressed neighborhoods [44-46]. These literatures are often separate, however, and the relative contribution of housing status and neighborhood characteristics on drug use or other health risking behaviors is not known. For example, it is not known to what extent the negative effects of neighborhood distress are mediated by residents' housing status. Similarly, research examining the potential moderating role of neighborhood characteristics on the association between housing status and health is needed.

Results from this study suggest that supportive and subsidized housing are still likely to be located in neighborhoods with high social disorder, and that neighborhood characteristics, particularly the visibility of drug sales, negatively impact the mental health of both housed and homeless participants. HUD could provide additional incentives to landlords to accept housing subsidies, for example providing tax incentives, to increase the attractiveness of accepting housing subsidies and reduce the tendency for subsidized and supportive housing recipients to be concentrated in the most distressed neighborhoods. Supportive housing in particular, should consider neighborhood drug and violent crime in locating their clients as high crime environments may reduce the benefits associated with providing stable housing and appropriate support services. Ultimately, however, interventions should target neighborhood-level risk factors. Potential approaches could include police and community partnerships to reduce neighborhood violence and the visibility of drug sales, or efforts to improve neighborhood cohesion and sense of efficacy to confront neighborhood problems. All participants in this study, although they were active drug users, wished to live in safe neighborhoods where they were not confronted daily with drug dealers and violence. It is time for public health researchers to both study the impact of the physical environment of cities on residents' health and develop effective interventions to address these.

There were a number of limitations to this study and results should be considered exploratory. First, data used in the geospatial analyses include only apartments for rent during a one-month period. It is possible that the apartments advertised and available differed from the rental market in Hartford at other points in time. However, subsequent newspaper searches indicate that the apartments advertised for rent during the study period were not atypical. In addition, we were unable to schedule interviews with all landlords and many landlords did not agree to participate in in-depth interviews. All but one of the landlords who agreed to be interviewed accepted housing subsidies, limiting the generalizability of the study. Interviewed landlords also were more likely to have unites with cheaper rents. While the sample is biased to those who accepted subsidies, all landlords interviewed described disincentives to accepting subsidies. Therefore, the barriers faced by drug users, even those with subsidies, are likely to be underestimated in this study. We were unable to complete interviews with 39% of the landlords or managers of the apartments listed for rent. This rate of non-completion is not unusual for phone interviews. We felt, however, that contacting landlords and managers who were advertising apartments for rent was the most accurate way to document the accessibility of free-market rental housing. Research studying where supportive housing residents and those who receive housing subsidies are able to live, and how neighborhood risk affects their drug use and HIV risk behaviors, is currently being carried out by our research team in Hartford.

## Competing interests

The authors declare that they have no competing interests.

## Authors' contributions

JDG conceptualized the design of the study, analyzed qualitative data and drafted the manuscript. EC geocoded and analyzed the geographic data, produced the figures and helped draft the paper. MC and HH conducted the brief telephone interviews, in-depth interviews with landlords and drug users, and helped analyze the qualitative data.
